# Swine Acute Diarrhea Syndrome Coronavirus Nucleocapsid Protein Antagonizes Interferon-*β* Production *via* Blocking the Interaction Between TRAF3 and TBK1

**DOI:** 10.3389/fimmu.2021.573078

**Published:** 2021-02-22

**Authors:** Zhihai Zhou, Yuan Sun, Jingya Xu, Xiaoyu Tang, Ling Zhou, Qianniu Li, Tian Lan, Jingyun Ma

**Affiliations:** ^1^ College of Animal Science, South China Agricultural University, Guangzhou, China; ^2^ Key Laboratory of Animal Health Aquaculture and Environmental Control, Guangzhou, China

**Keywords:** SADS-CoV, nucleocapsid protein, TANK binding kinase 1, interferon beta, TRAF3

## Abstract

Swine acute diarrhea syndrome coronavirus (SADS-CoV), first discovered in 2017, is a porcine enteric coronavirus that can cause acute diarrhea syndrome (SADS) in piglets. Here, we studied the role of SADS-CoV nucleocapsid (N) protein in innate immunity. Our results showed that SADS-CoV N protein could inhibit type I interferon (IFN) production mediated by Sendai virus (Sev) and could block the phosphorylation and nuclear translocation of interferon regulatory factor 3 (IRF3). Simultaneously, the IFN-*β* promoter activity mediated by TANK binding kinase 1 (TBK1) or its upstream molecules in the RLRs signal pathway was inhibited by SADS-CoV N protein. Further investigations revealed that SADS-CoV N protein could counteract interaction between TNF receptor-associated factor 3 (TRAF3) and TBK1, which led to reduced TBK1 activation and IFN-*β* production. Our study is the first report of the interaction between SADS-CoV N protein and the host antiviral innate immune responses, and the mechanism utilized by SADS-CoV N protein provides a new insight of coronaviruses evading host antiviral innate immunity.

## Introduction

Swine acute diarrhea syndrome coronavirus (SADS-CoV) is a newly discovered porcine enteric coronavirus, causing acute diarrhea and vomiting, with a nearly 90% fatal mortality in piglets resulted in large economic losses for the pig-breeding industry in China (1–5). As a single-stranded positive-sense RNA virus, SADS-CoV belongs to the genus *Alphacoronavirus* of the family *Coronaviridae* (1–3). The genome of SADS-CoV is approximately 27 kb with the typical gene order of coronaviruses, which contains open reading frame (ORF) 1a located from the 5′ end, followed by ORF1b, spike (S), one accessory gene NS3a, envelope (E), membrane (M), nucleocapsid (N) and two accessory genes NS7a and NS7b (1).

Antiviral innate immunity is the host first-line defense to fight viral infections. During coronavirus infection, the replicative intermediate such as nucleic acid produced by viruses in infected cells that act as pathogen-associated molecular patterns (PAMPs) could be recognized by host pattern recognition receptors (PRRs) (6). Retinoic acid-inducible gene I (RIG-I) and Melanoma differentiation-associated gene 5 (MDA5) are critical PRRs in the cytoplasm of host cells for recognizing viral dsRNA (7, 8). After recognizing the cytoplasmic dsRNA, RIG-I and/or MDA5 is activated and interacts with the CARD region of interferon-beta (IFN-*β*) promoter stimulator through their caspase activation and recruitment domain region, stimulating the downstream TANK binding kinase 1 (TBK1) and inhibitor of *κ*B kinase-*ϵ* (IKK*ϵ*) (9, 10). Generally, the activation of TBK1 by IPS-1 is a complex process, which requires recruiting the pre-association complex TBK1-TRAFs (TRA2/3/6) to approach IPS-1 for TBK1 autophosphorylation  (9, 10). Activated TBK1 leads phosphorylation and nuclear translocation of IRF3 and NF-*κ*B, which can induce type I interferon (IFN-I) production (11–14).

Accordingly, coronaviruses have evolved various strategies to antagonize the host innate immunity. In particular, multiple proteins encoded by coronaviruses play important roles in IFN evasion by targeting IFN itself or IFN-associated signal molecules. The N protein encoded by coronavirus has the highest abundance in infected cells and is essential for viral transcription and assembly (15–17). In addition to viral function, the N protein is considered an important IFN antagonist, for example, porcine epidemic diarrhea virus (PEDV), porcine delta coronavirus (PDCoV), severe acute respiratory syndrome coronavirus (SARS-CoV), and mouse hepatitis virus (MHV) N protein suppressing IFN-*β* production through different mechanisms (18–20). Besides the N protein, other structural or non-structural proteins of coronaviruses, including middle east respiratory syndrome coronavirus (MERS-CoV) M, ORF4a, ORF4b, and ORF5 (21–23), PEDV 3C-like protease (24), and PDCoV NS6 (25) also have functions in antagonizing IFN-*β* expression. Although our previous study has shown that SADS-CoV could inhibit IFN-*β* production by targeting IPS-1 (26), the evasion roles of proteins encoded by SADS-CoV in antiviral innate immunity have not been reported, as well as the molecular mechanisms through which SADS-CoV proteins modulate IFN-*β* expression are yet to be elucidated.

In the present study, we investigated the role of SADS-CoV N protein in regulating the host innate immune response. We found that SADS-CoV N protein was an IFN-*β* antagonist. Mechanistically, SADS-CoV N protein inhibited IFN-*β* production by targeting TBK1 to disturb the interaction between TRAF3 and TBK1. Compared with other coronavirus N protein, the strategy of SADS-CoV N protein to counteract antiviral innate immunity is a novel mechanism.

## Materials and Methods

### Viruses, Cells, and Reagents

The Human embryonic kidney cells (HEK-293T) and swine testicular (ST) cells were preserved in the Key Laboratory of Animal Health Aquaculture and Environmental Control, South China Agricultural University, and were supplemented in Dulbecco’s modified Eagle’s medium (DMEM) with 10 % fetal bovine serum (FBS) at 37 °C in a humidified 5% CO_2_ incubator. Sendai virus (Sev) was kindly provided by the Wuhan Institute of Virology, Chinese Academy of Sciences. The Dual-Luciferase^®^ Reporter Assay System was purchased from Promega Corporation (Madison, WI, USA). Anti-IRF3, anti-phosphorylated IRF-3 (p-IRF-3), anti-TBK1, anti-phosphorylated TBK1 (p-TBK1), and anti-*β*-actin antibodies were purchased from Abcam (UK). Monoclonal antibodies against IRF3, IPS-1, TRAF3, TBK1, FLAG, HA, and MYC were obtained from Proteintech (Wuhan, China). Goat anti-rabbit IgG H&L (HRP), goat anti-mouse IgG H&L (HRP), goat anti-mouse IgG H&L (Alexa Fluor^®^ 488), and goat anti-rabbit IgG H&L (Alexa Fluor^®^ 594) were purchased from Absin Bioscience, Inc. (Shanghai, China). IPKine HRP, Mouse Anti-Rabbit IgG LCS, and IPKine HRP, Goat Anti-Mouse IgG LCS (avoid IgG heavy chain interference) were purchased from FangYuan biotechnology company (Guangzhou, China).

### Plasmids

The plasmids IFN-*β*-Luc for IFN-*β*, IRF3-Luc for IRF3, NF-*κ*B-Luc for NF-*κ*B and internal control plasmid pRL-TK were kindly donated by Dr. Shaobo Xiao (Huazhong Agricultural University, Wuhan, Hubei Province, China). SADS-CoV N protein expression plasmids pCMV-FLAG-N and MYC-N were previously constructed by our laboratory. The expression plasmids human FLAG-RIG-I and FLAG-MDA5 were kindly donated by Harbin Veterinary Research Institute. The recombinant expression plasmid human HA-IPS-1, HA-IKK*ϵ*, HA-TBK1, MYC-TBK1, HA-TRAF3, HA-IRF3, and porcine HA-TRAF3, MYC-TBK1 were constructed by the Miaoling biotechnology company (Wuhan, China).

### Luciferase Reporter Gene Assay

HEK-293T cells and ST cells reaching approximately 80% confluence in 24-well plates were co-transfected with increasing amounts of pCMV-FLAG-N expression plasmids and the reporter plasmid (IFN-*β*-Luc, IRF3-Luc, and NF-*κ*B-Luc) of 0.1 μg/well together with internal control plasmid pRL-TK of 0.01 μg/well. After 24 h transfection, cells were treated with Sev as a positive control for an additional 12 h. Or HEK-293T cells were co-transfected with luciferase reporter plasmids IFN-*β*-Luc and internal control plasmid pRL-TK together with the expression plasmids (0.5 μg) of the molecules in the RLRs’ signaling pathway (RIG, MDA-5, IPS-1, TRAF3, IKK*ϵ*, TBK1, and IRF3) for 28 h. Harvested cells were lysed by Passive Lysis Buffer (PLB), and activities of the firefly luciferase and Renilla luciferase were determined by Bio-Tek Synergy Neo2 according to the Dual-Luciferase reporter assay system (Promega). Data were expressed as the relative firefly luciferase activities normalized to Renilla luciferase activities from three independently conducted experiments.

### RNA Extraction and Quantitative Real-Time RT-qPCR

HEK-293T cells grown in 6-well plates were transfected with pCMV-FLAG-N of 2 μg/well for 24 h, then cells were infected with Sev as a positive control for an additional 12 h. Total RNA was extracted from the transfected cells using TRIzol reagent (Invitrogen, USA) and was reverse-transcribed into complementary DNA (cDNA) by using the PrimeScript™ RT reagent Kit with gDNA Eraser (Takara, Biotechnology, Dalian, China). The cDNA was then used as the template in an SYBR green PCR assay (Genstar, Biotechnology, Beijing, China) with specific primer pairs targeting IFN-*β* or Sev HN and GAPDH (**Table 1**). The abundance of the individual mRNA transcript in each sample was assayed three times and normalized to GAPDH mRNA (the internal control).

**Table 1:** Primers used for real-time RT-PCR.

Primers	Sequence (5′–3′)	References
IFN-β	F:TCTTTCCATGAGCTACAACTTGCT	(18)
	R:GCAGTATTCAAGCCTCCCATTC	
GAPDH	F:TCATGACCACAGTCCATGCC	(18)
	R:GGATGACCTTGCCCACAGCC	
SEV-HN	F:AAAATTACATGGCTAGGAGGGAAAC	(25)
	R:GTGATTGGAATGGTTGTGACTCTTA	

### ELISA Assay for IFN-*β* Protein

HEK-293T cells were transfected with pCMV-FLAG-N for 24 h, then stimulated with Sev for another 12 h. The supernatants were harvested to measure the secretion of IFN-*β* using the Human IFN-*β* ELISA kit according to the manufacturer’s instructions (Cusabio, Wuhan, China).

#### Indirect Immunofluorescence Assay

When the confluency of HEK-293T cells reached approximately 80% in laser confocal dishes, cells were transfected with pCMV-FLAG-N and jetPRIME^®^ transfection reagent for 24 h, then infected with Sev as a positive control for an additional 10 h. Treated cells were fixed with 4% paraformaldehyde for 15 min and then permeated with 0.1% Triton X-100 for 10 min. After three times of washing by phosphate-buffered saline (PBS), cells were sealed with PBS containing 5% bovine serum albumin (BSA) for 1 h and then incubated separately with rabbit polyclonal antibody against IRF3 or TBK1 and mouse polyclonal antibody against the FLAG for 1 h at room temperature. Then cells were processed with goat anti-mouse IgG H&L (Alexa Fluor^®^ 488) and goat anti-rabbit IgG H&L (Alexa Fluor^®^ 594) for 1 h followed by 4′,6-diamidino-2-phenylindole-dihydrochloride (DAPI) for 15 min at room temperature. After washing with PBS, cells were added with an anti-fluorescence quenching agent and examined with the confocal laser scanning microscope (Leica SPE; Leica, Germany) to collect fluorescent images.

### Western Blot Analysis

HEK-293T cells and ST cells cultured in 6-well plates were treated accordingly to indicative times, lysed in RIPA Lysis Buffer supplemented with a protease inhibitor cocktail, and boiled for 10 min, or samples being first processed by immunoprecipitation were added with the appropriate amount of 5*SDS-PAGE Sample Loading Buffer to make 1*SDS loading buffer and boiled for 10 min. The lysates were separated by 10% sodium dodecyl sulfate-polyacrylamide gel electrophoresis (SDS-PAGE) and transferred onto Immobilon-P membrane (EMD Millipore, Billerica, MA, USA). After being blocked with QuickBlock™ Blocking Buffer for Western Blot (Boytime), the membrane was incubated with rabbit monoclonal antibodies against TBK1, p-TBK1, IRF-3, p-IRF-3, IPS-1, FLAG, and HA at room temperature for 1 h, followed by incubation with horseradish peroxidase (HRP)-conjugated anti-rabbit IgG (1:10,000) for 30 min at room temperature. The expression of *β*-actin was detected with an anti-*β*-actin mouse monoclonal antibody to confirm the loading of equal protein amounts. Protein blots were detected using enhanced chemiluminescence (ECL) detection system and an Azure c600 visible fluorescent western blot imaging system (Azure Biosystems, America).

### Co-Immunoprecipitation Analysis

HEK-293T cells and ST cells grown in 60-mm dishes were transfected with expression plasmids, and the whole-cell lysates were collected by Cell lysis buffer (Boytime) for Western blot and immunoprecipitation; that is, samples were immunoprecipitated with the protein A/G magnetic beads and incubated with the antibody for 4 h at 4°C, or immunoprecipitated with Anti-Flag Affinity Gel overnight at 4°C. After three times of washing by phosphate-buffered saline and Tween 20 (PBST), the anti-Flag affinity gel or protein A/G magnetic beads were added with 50 ul 1*SDS-PAGE Sample Loading Buffer and boiled for 5 min at 100°C. The levels of protein expression after immunoprecipitation were then analyzed by Western blot.

### Statistical Analysis

Unless otherwise specified, all data were presented as mean ± SEM of independent experiments in triplicate. The software SPSS statistics 22 (International Business Machines Corporation, America) was employed for statistical analyses. Comparisons between groups were considered statistically significant at p < 0.05. Student’s t-test was used for determining statistical significance, and *P*-values of <0.05 were considered statistically significant.

## Results

### SADS-CoV N Protein Inhibits Sev-mediated IFN-*β* Expression

**Figure 1:**
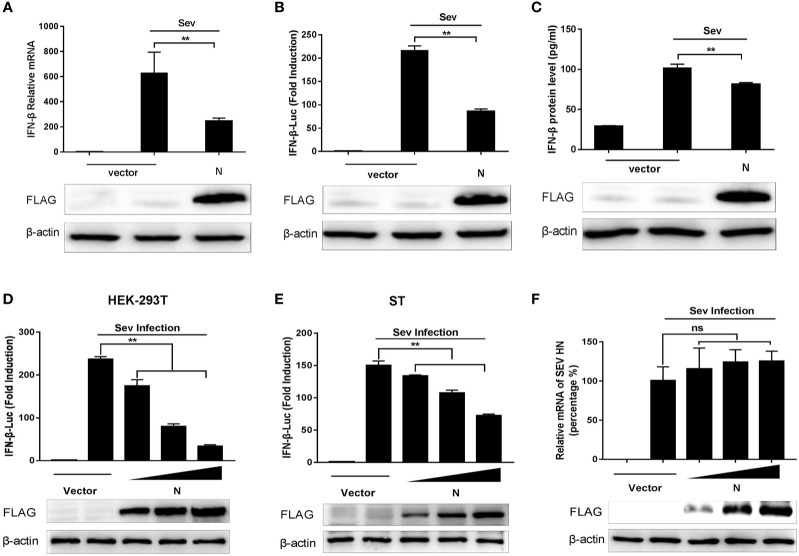
SADS-CoV N protein inhibits Sev-mediated IFN-*β* expression. **(A)** HEK-293T cells cultured in 6-well plates were transfected with pCMV-FLAG-N expression plasmids of 2 μg/well. After 24 h, cells were stimulated with Sev for an additional 12 h. And then, total RNA, lysed products, and supernatants were collected separately. The mRNA levels of IFN-*β* were evaluated by quantitative real-time RT-qPCR. **(B)** Promoter activities of IFN-*β*-Luc were analyzed by Dual-Luciferase reporter assay. The relative firefly luciferase activity was normalized to the Renilla luciferase activity, and the value of untreated empty vector was set as 1. **(C)** The IFN-*β* protein levels were analyzed by IFN beta Human ELISA Kit. HEK-293T cells **(D)** or ST cells **(E)** were co-transfected with the reporter plasmid (IFN-*β*-Luc) of 0.1 μg/well and internal control plasmid pRL-TK of 0.01 μg/well together with increasing quantities (0.25, 0.5, or 1 μg) of pCMV-FLAG-N expression plasmids for 24 h, and then cells were infected with Sev. The relative activity of the IFN-*β* promoter was determined by Dual-luciferase assay. **(F)** HEK-293T cells cultured in 6-well plates were transfected with increasing quantities of pCMV-FLAG-N expression plasmids. After 24 h, cells were stimulated with Sev for an additional 12 h. The total RNA was extracted, and the mRNA expression of the Sev HN gene was evaluated by quantitative real-time RT-qPCR. The expression of SADS-CoV N protein with anti-FLAG antibody was detected by Western blot, and anti-*β*-actin antibody expression was used as the loading control. **P < 0.01; ns, nonsignificant differences in data.

According to previous studies, Sev is considered to be a strong inducer of the RIG-I-like receptor (RLR)-mediated IFN signaling pathway (27, 28). To investigate whether SADS-CoV N protein could inhibit the expression of IFN-*β*, HEK-293T cells were transfected with SADS-CoV-N protein-expressing plasmid (pCMV-FLAG-N) or empty plasmid for 24 h and infected with Sendai virus (Sev) for another 12 h. Then the production of IFN-*β* was analyzed. Results showed that the mRNA expression, the promoter activity, and the protein production of IFN-*β* induced by Sev in HEK-293T cells were significantly inhibited by SADS-CoV N protein (**Figures 1A–C**). To further confirm these results, ST cells, pig-derived cell line, were also subjected to SADS-CoV infection. ST and HEK-293T cells were co-transfected with increasing quantities of plasmids pCMV-FLAG-N or empty vector and luciferase reporter system (IFN-*β*-Luc and pRL-TK), then stimulated with Sev for an additional 12 h. As expected, the N protein of SADS-CoV had a dose-dependent inhibitory effect on IFN-*β* promoter activation induced by Sev in HEK-293T and ST cells (**Figures 1D, E**). To verify whether the N protein could affect Sev’s proliferation, we used RT-qPCR to detect the gene expression of Sev HN in HEK-293T cells transfected with pCMV-FLAG-N. The results showed no significant difference in mRNA expression of the Sev HN gene in transfected cells and in mock-transfected cells (**Figure 1F**), which indicated that the proliferation of Sev was not blocked by SADS-CoV N protein and further demonstrated the inhibition of N protein on IFN-*β* expression. In a word, these results revealed that N protein encoded by SADS-CoV was an antagonist of IFN-*β*.

### SADS-CoV N Protein Impedes Sev-Induced Phosphorylation and Nuclear Translocation of IRF-3 and NF-κB

**Figure 2:**
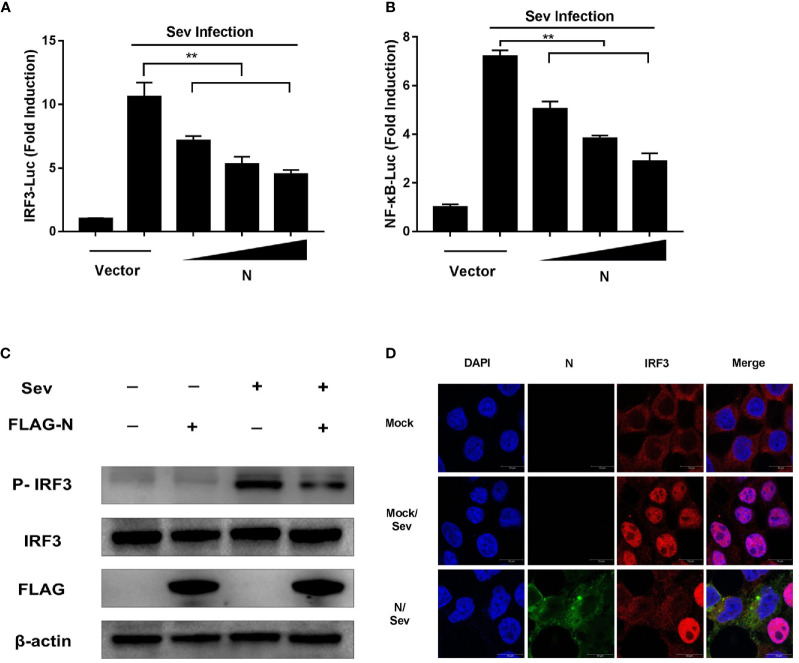
SADS-CoV N protein inhibits Sev-induced phosphorylation and nuclear translocation of IRF-3. HEK-293T cells were co-transfected with pCMV-FLAG-N expression plasmids and the luciferase reporter IRF3-Luc **(A)** or NF-*κ*B-Luc **(B)** together with pRL-TK for 24 h and then stimulated with Sev for 12 h. Cell lysates were collected, and the promoter activity of IRF3 or NF-*κ*B was analyzed by Dual-luciferase assay. **(C)** pCMV-FLAG-N expression plasmids were transfected with HEK-293T cells. After 24 h transfection, cells were treated with Sev for another 12 h. Cell lysates were collected for Western blot analysis with anti-phosphorylated IRF3 (Ser396), anti-IRF3, anti-FLAG, or anti-*β*-actin antibodies. **(D)** HEK-293T cells were transfected with pCMV-FLAG-N expression plasmids. After 24 h transfection, cells were treated with Sev for another 8 h. After cell fixation and permeation, an indirect immunofluorescence assay was analyzed with rabbit anti-IRF3 and mouse anti-FLAG antibodies. The confocal laser scanning microscope (Leica SPE; Leica, Germany) was used to detect signals of nuclear translocation of IRF3 (red) and SADS-CoV N protein (green). **P < 0.01.

The activations of transcription factors IRF3 and NF-*κ*B are essential for IFN-*β* production. To explore the effect of SADS-CoV N protein on the activation of IRF3 and NF-*κ*B, HEK-293T cells were co-transfected with increasing quantities of plasmids pCMV-FLAG-N and the luciferase reporter system (pRL-TK and IRF3-Luc or NF-*κ*B-Luc luciferase reporter plasmids). At 24 h post-transfection, cells were subjected to Sev infection for 12 h. As shown in **Figure 2**, the promoter activities of IRF3 (**Figure 2A**) or NF-*κ*B (**Figure 2B**) induced by Sev were significantly inhibited by overexpression of SADS-CoV N protein in a dose-dependent manner.

IRF3 is a critical interferon regulatory factor, and its activation can drive IFN-*β* transcription. The activation hallmarks of IRF3 are phosphorylation and nuclear translocation (12). To further explore how SADS-CoV N protein block IRF3 promoter activity, the phosphorylation and nuclear translocation of IRF3 in HEK-293T cells treated with pCMV-FLAG-N together with Sev were analyzed by Western blot and confocal microscopy assay, respectively. As shown in **Figure 2C**, compared with that in uninfected cells, the phosphorylation of IRF3 was significantly promoted by Sev infection. However, the phosphorylation of IRF3 induced by Sev was inhibited by SADS-CoV N protein. Similar to the result of Western blot, the confocal microscopy assay showed that the nuclear translocation of IRF3 induced by Sev was also impeded by N protein encoded by SADS-CoV (**Figure 2D**). Altogether, these results clearly showed that SADS-CoV N protein could inhibit the activation of IRF-3 by blocking its phosphorylation and nuclear translocation.

### SADS-CoV N Protein Suppresses the RLRs’ Signaling Pathway

**Figure 3:**
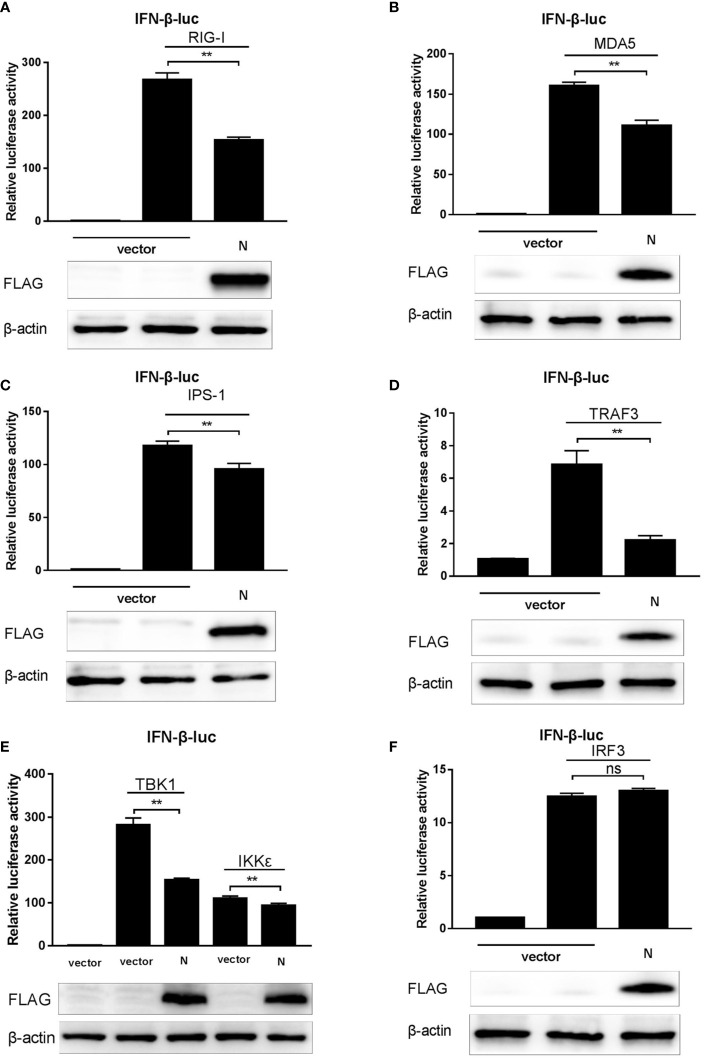
SADS-CoV N protein suppresses the RLRs signaling pathway. **(A–F)** pCMV-FLAG-N and a series of expression plasmids encoding the RLRs’ signaling pathway adaptors (RIG-I, MDA5, IPS-1, TRAF3, IKK*ϵ*, TBK1, and IRF3), together with a luciferase reporter plasmid IFN-*β*-Luc and the internal control plasmid pRL-TK were co-transfected to HEK-293T cells. After 24 h, cell lysates were collected using a passive lysis buffer, and the relative activity of the IFN-*β* promoter was measured with the Dual-luciferase assay. Or cell protein lysates were collected using RIPA lysis buffer, and the expression of SADS-CoV N protein and *β*-actin (as the loading control) was detected by Western blot. **P < 0.01; ns, nonsignificant differences in data.

Coronaviruses are considered to interrupt IFN-*β* expression mainly through blocking the RLRs-mediated signaling pathway (26, 29, 30). Our data showed that SADS-CoV N protein was an antagonist of IFN-*β* (**Figure 1**) and could inhibit the Sev-mediated activation of IRF3 (**Figure 2**), which suggested that the N protein may also act on the RLR-mediated signaling pathway. To confirm this speculation, a series of expression plasmids encoding the molecules of the RLRs’ signaling pathway, including RIG-I, MDA-5, IPS-1, TRAF3, IKK*ϵ*, TBK1, IRF3, and expression plasmids of pCMV-FLAG-N, together with the luciferase reporter system (pRL-TK and IFN-*β*-Luc) were co-transfected to HEK-293T cells. After 24 h, cell lysates were harvested to analyze the activity of the IFN-*β* promoter. The results showed that the overexpression of molecules in the RLRs’ signaling pathway mentioned above all led to the significant increase of the activity of IFN-*β* promoter, but in cells co-transfected with the plasmid of pCMV-FLAG-N, the activity of IFN-*β* promoter induced by RIG-I, MDA5, IPS-1, TRAF3, IKK*ϵ*, and TBK1 was inhibited by SASD-CoV N protein (**Figures 3A–E**). While the overexpressing of pCMV-FLAG-N failed to block the IRF3-mediated production of IFN-*β* (**Figure 3F**). These results indicated that SADS-CoV N protein interacted with host adaptor molecules to inhibit IFN-*β* production, and TBK1/IKK*ϵ* could be the target molecules. Based on these findings, we speculated that SADS-CoV N protein could block signal transmission from TBK1/IKK*ϵ* to IRF3 to impair the expression of IFN-*β*.

### SADS-CoV N Protein Interacts With TBK1 and IKK*ϵ*

**Figure 4:**
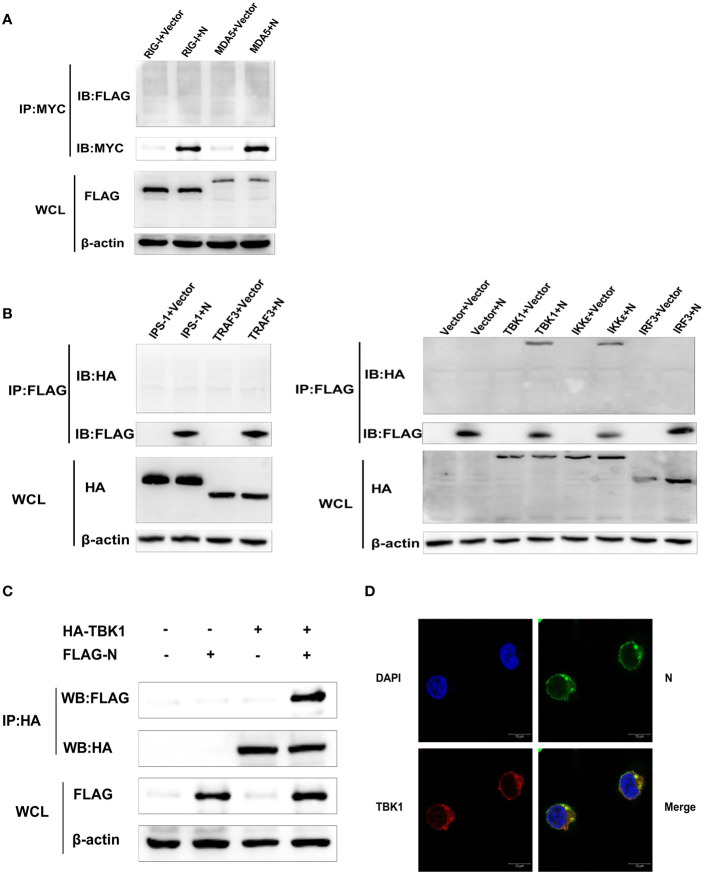
SADS-CoV N protein interacts with TBK1. **(A)** HEK-293T cells were transfected with pcDNA-MYC-N along with Flag-RIG-I or Flag-MDA5. Then protein expression levels were analyzed by Western blot. **(B)** Or HEK-293T cells were transfected with pCMV-FLAG-N and a series of expression plasmids encoding the molecules of the RLRs’ signaling pathway (HA-IPS-1, HA-TRAF3, HA-IKKϵ, HA-TBK1, and HA-IRF3). The whole-cell lysates were collected and immunoprecipitated with the anti-Flag affinity gel or anti-MYC beads at 28 h post-transfection. Then protein expression levels were analyzed by Western blot. **(C)** HEK-293T cells were co-transfected with plasmids HA-TBK1 and plasmids pCMV-FLAG-N. After 28 h post cotransfection, the whole cell lysates and immunoprecipitated with the protein A/G magnetic beads containing anti-HA antibody. Then protein expression levels were analyzed by Western blot. **(D)** HEK-293T cells were co-transfected with plasmids HA-TBK1 and plasmids pCMV-FLAG-N. After 28 h cotransfection, IFA was used to analyze TBK1 protein and N protein’s subcellular localization in HEK-293T cells by confocal laser scanning microscope (Leica SPE; Leica, Germany). Anti-HA antibody for detecting TBK1 (red), anti-FLAG antibody for detecting SADS-CoV N protein (green). The location of the nucleus was stained with DAPI (blue).

To further identify the potential target for SASD-CoV N protein of the RLRs signaling pathway, HEK-293T cells were transfected with pCMV-FLAG-N or pcDNA-MYC-N along with a series of expression constructs encoding the molecules of the human RLRs’ signaling pathway (Flag-RIG-I, Flag-MDA5, HA-IPS-1, HA-TRAF3, HA-IKK*ϵ*, HA-TBK1, and HA-IRF3). At 28 h post-transfection, the whole-cell lysates were collected for immunoprecipitation assay with the anti-Flag affinity gel or anti-MYC protein A/G magnetic beads. The results showed that only HA-TBK1 and HA-IKK*ϵ* proteins were detected in Flag-N precipitant (**Figures 4A, B**), indicating an interaction between SADS-CoV N protein and TBK1/IKK*ϵ*.

Because the activity of IFN-*β* promoter induced by TBK1 was higher than that induced by IKK-*ϵ* in this study, and the blocking effect of SADS-CoV N protein on the activation of IFN-*β* promoter induced by TBK1 was more significant than that induced by IKK-*ϵ* (**Figure 3E**), TBK1 was chosen to be the focus of our following investigations. To confirm the interaction between SADS-CoV N protein and TBK1, HEK-293T cells were co-transfected with plasmids HA-TBK1 and pCMV-FLAG-N, and 28 h later, the lysates were immunoprecipitated with the protein A/G magnetic beads containing anti-HA antibody. As shown in **Figure 4C**, HA-TBK1 could pull down SADS-CoV N protein, which further verified the interaction between TBK1 and SADS-CoV N protein. Next, the localization of SADS-CoV N protein and TBK1 in cells were analyzed to investigate whether there was a foundation for their interaction. HEK-293T cells were co-transfected with plasmids HA-TBK1 and plasmids pCMV-FLAG-N. After 28 h cotransfection, IFA was applied to analyze TBK1 and N protein’s subcellular localization. As shown in **Figure 4D**, both SADS-CoV N protein and TBK1 protein located in the cytoplasm provided a further basis for the interaction between N protein and TBK1.

### SADS-CoV N Protein Disrupts the Interaction Between IPS-1 and TBK1

**Figure 5:**
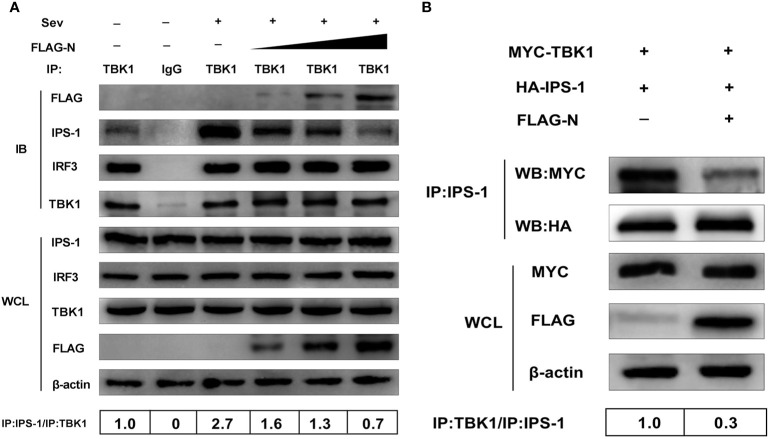
SADS-CoV N protein disrupts the interaction between IPS-1 and TBK1. **(A)** HEK-293T cells were transfected with increasing quantities of plasmids pCMV-FLAG-N or mock-transfected for 24 h, and the cells were treated with Sev for an additional 12 h. The whole-cell lysates were collected and immunoprecipitated with the protein A/G magnetic beads containing the anti-TBK1 antibody or normal rabbit IgG. **(B)** HEK-293T cells were co-transfected with expression plasmids MYC-TBK1 and HA-IPS-1 and pCMV-FLAG-N or empty vector for 28 h. The whole-cell lysates were collected and immunoprecipitated with the protein A/G magnetic beads containing anti-IPS-1 antibody. Then protein expression levels were analyzed by Western blot with anti-FLAG, anti-MYC, anti-HA, anti-IPS-1, anti-TBK1, anti-IRF3, and anti-*β*-actin monoclonal antibodies (MAbs), respectively. Levels of IPS-1 or TBK1 after immunoprecipitation were quantitated densitometrically and corrected with TBK1 or IPS-1 levels in immunoprecipitation, and the ratio between the immunoprecipitated and co-immunoprecipitated proteins in untreated cells was set as 1.0.

It has been reported that IPS-1 could activate the transcription factor IRF3 through TBK1/IKK*ϵ* after RNA virus infection (31, 32). Therefore, the interaction between IPS-1 and TBK1 or IRF3 and TBK1 is crucial for signal transduction. To determine whether SADS-CoV N protein inhibits the association between IPS-1 and TBK1 or IRF3 and TBK1, HEK-293T cells were transfected with increasing quantities of plasmids pCMV-FLAG-N or mock-transfected. Then the cells were treated with Sev. The whole-cell lysates were collected and immunoprecipitated with the protein A/G magnetic beads containing anti-TBK1 or normal rabbit IgG. The protein expression levels were analyzed by Western blot with anti-FLAG, anti-IPS-1, anti-TBK1, and anti-IRF3 monoclonal antibodies (MAbs), respectively. As shown in **Figure 5A**, IPS-1 protein was detected in TBK1 precipitates.

Simultaneously, the interaction between IPS-1 and TBK1 was significantly enhanced by Sev infection but was inhibited by the N protein. With the increased dose of the N protein transfection, the IPS-1 protein level detected by immunoprecipitation decreased gradually. Notably, IRF3 was also effectively pulled down by TBK1, but the coprecipitated IRF3 protein was not affected by overexpressed N protein. These results suggested that the N protein disturbed TBK1 and IPS-1, not interactions between TBK1 and IRF3. To further confirm this result, HEK-293T cells were co-transfected with expression plasmids MYC-TBK1 and HA-IPS-1 and pCMV-FLAG-N or an empty vector. The lysates were collected and immunoprecipitated with the protein A/G magnetic beads containing anti-IPS-1 antibody. Consistent with the results mentioned above, TBK1 was effectively precipitated with IPS-1, and the interaction between IPS-1 and TBK1 was inhibited by the N protein, too (**Figure 5B**). In short, these results indicated that SADS-CoV N protein blocked the IPS-1–TBK1 interaction, and its inhibitory effect was a dose-dependent manner.

### SADS-CoV N Protein Impairs Interaction Between TBK1 and TRAF3

**Figure 6:**
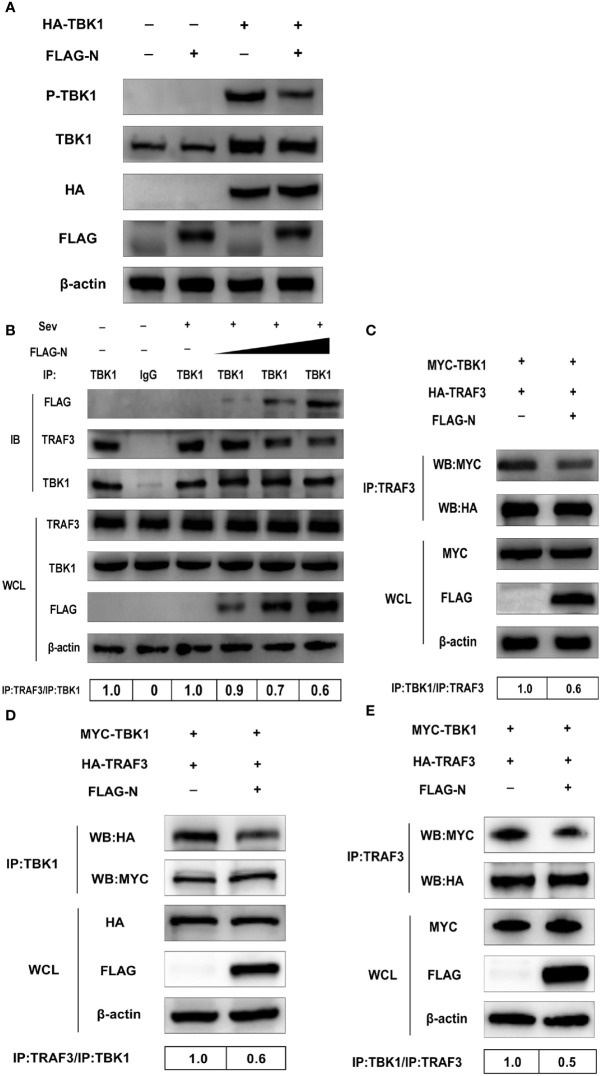
SADS-CoV N protein disrupts the interaction between TBK1 and TRAF3. **(A)** HEK-293T cells were co-transfected with expression plasmids pCMV-FLAG-N and HA-TBK1 or mock-transfected for 24 h. The whole-cell lysates were collected and analyzed by Western blot with anti-FLAG, anti-HA, anti-TBK1, and anti-phosphorylated TBK1 monoclonal antibodies (MAbs), respectively. **(B)** HEK-293T cells were transfected with plasmids pCMV-FLAG-N with increasing quantities or mock-transfected for 24 h, and cells were treated or untreated with Sev for an additional 12 h. The whole-cell lysates were collected and immunoprecipitated with the protein A/G magnetic beads containing the anti-TBK1 antibody or normal rabbit IgG. **(C)** HEK-293T cells were co-transfected with expression plasmids MYC-TBK1 and HA-TRAF3 and pCMV-FLAG-N or empty vector for 28 h. The lysates were collected and immunoprecipitated with the protein A/G magnetic beads containing anti-TRAF3 antibody. ST cells were co-transfected with expression plasmids of porcine MYC-TBK1 and porcine HA-TRAF3 and pCMV-FLAG-N or empty vector for 28 h. The whole-cell lysates were collected and immunoprecipitated with the protein A/G magnetic beads containing anti-TBK1 antibody **(D)** or anti-TRAF3 **(E)**. The protein expression levels were analyzed by Western blot with anti-FLAG, anti-TRAF3, anti-TBK1, anti-MYC, anti-HA, and anti-*β*-actin monoclonal antibodies (MAbs), respectively. Levels of TRAF3 or TBK1 after immunoprecipitation were quantitated densitometrically and corrected with TBK1 or TRAF3 in immunoprecipitation, and the ratio between the immunoprecipitated and co-immunoprecipitated proteins in untreated cells was set as 1.0.

Previous studies indicate that IPS-1 recruits and activates TBK1 *via* pre-associated TRAFs-TBK1 complex after RNA virus infection, and the phosphorylation is the sign of TBK1 activation (9, 10). Based on our data mentioned above, we speculated that SASD-CoV N protein could disrupt the phosphorylation of TBK1 induced by itself. To test our hypothesis, the expression plasmid pCMV-FLAG-N and HA-TBK1 were co-transfected into HEK-293T cells for 28 h. The phosphorylation level of TBK1 was detected by Western blot with anti-phosphorylation TBK1 (p-TBK1) antibody. As shown in **Figure 6A**, the overexpression of TBK1 significantly increased the protein level of p-TBK1, which was also inhibited by the overexpressing of the N protein. This result indicated that the N protein impaired the level of p-TBK1 induced by TBK1, so we further supposed that SADS-CoV N protein may disrupt the activation of TBK1 through affecting the formation of the TRAFs–TBK1 complex.

To investigate the hypothesis, HEK-293T cells were transfected with the plasmid pCMV-FLAG-N with increasing quantities or mock-transfected. Then cells were treated or untreated with Sev. The whole-cell lysates were collected and immunoprecipitated with the protein A/G magnetic beads containing the anti-TBK1 antibody or normal rabbit IgG. Then protein expression levels were analyzed by Western blot with anti-FLAG, anti-TRAF3, and anti-TBK1 monoclonal antibodies (MAbs). The results showed that TRAF3 was effectively precipitated by TBK1, but was inhibited by the N protein in a dose-dependent manner, indicating that the N protein disturbed the interaction between TBK1 and TRAF3 (**Figure 6B**). To further determine the N protein effect on the interaction between TBK1 and TRAF3, we also tested whether N protein could prevent TRAF3 from pulling down TBK1. As shown in **Figure 6C**, TBK1 was initially effectively pulled down by TRAF3, but the degree of pulldown was also weakened by the N protein, which further proved inhibitory of the N protein on the interaction between TBK1 and TRAF3. As SADS-CoV was a porcine virus, to verify the results obtained from HEK-293T cells, we chose ST cells in the subsequent study using the TRAF3 antibody and TBK1 antibody, respectively, for co-immunoprecipitation assay. Following findings in HEK-293T cells, SADS-CoV N protein blocked the pulldown between TRAF3 and TBK1 (**Figures 6D, E**), indicating that the N protein also inhibits interactions between TBK1 and TRAF3 in ST cells. Altogether, these results determined that SADS-CoV N protein disturbed interactions between TRAF3 and TBK1 in a dose-dependent manner.

## Discussion

In recent years, the N protein of coronavirus has attracted increasing attention, especially the N protein strategy to counteract the host innate immunity (33, 34). For the first time, we investigated the role of SADS-CoV N protein in the host antiviral immune response. Our results showed that the N protein of SADS-CoV could antagonize the production of IFN-*β* in the way of blocking the activation of TBK1 through disrupting the interaction between TRAF3 and TBK1, which is a novel mechanism utilized by the N protein to help the virus escape the host innate immunity to the best of our knowledge.

Previous studies about interactions between coronaviruses and host innate immune responses have indicated that coronaviruses, such as PEDV (29), SARS-CoV (35), MERS-CoV (22), and PDCoV (36) mainly focus on the RLRs signaling pathway to interrupt IFN-*β* expression. The results in our study showed that SADS-CoV N protein could inhibit not only Sev-mediated activation of IRF3 but also block IFN-*β* production induced by RIG-I, MDA-5, IPS-1, TRAF3, IKK*ϵ*, and TBK1, which suggested that SADS-CoV N protein also disrupted IFN-*β* expression by acting on the RLRs signaling pathway. And in this pathway, after RIG-I/MDA5 recognizing the pathogen-associated molecular patterns, signals would be transmitted to the downstream through the IPS-1–TBK1–IRF3 signal axis (9, 27, 31). Accordingly, coronaviruses have developed strategies to target this signal axis to inhibit IFN-*β* production (18, 37, 38). Our analysis revealed that the N protein of SADS-CoV inhibited the interaction between IPS-1 and TBK1 from counteracting the IPS-1-TBK1 signal axis transmission.

It has been reported that TBK1/IKK*ϵ* is activated by recruitment to IPS-1 through a pre-association with TRAFs (TRA2/3/6) (9, 10), indicating that the pre-associated TRAFs–TBK1/IKK*ϵ* complex is very important for IPS-1–TBK1 signal axis signal transmission. Our further investigation clearly showed that SADS-CoV N protein could disrupt the interaction between TRAF3 and TBK1, but not between TRAF6/TRAF2 and TBK1 (data not shown), which demonstrated that SADS-CoV N protein antagonized IFN-*β* production *via* disturbing the formation of TRAF3–TBK1 complex to counteract the interaction between IPS-1 and TBK1. Although this is a new mechanism utilized by the N protein of the coronavirus, a similar strategy has been found in the M protein of coronavirus. It was reported that MERS-CoV M could antagonize the production of IFN-*β* by obstructing the interaction between TBK1 and TRAF3 (38). It should be noted that the interference effect of MERS-CoV M protein is based on the interaction between the protein and TRAF3 rather than TBK1, which is different from that of SADS-CoV N protein. The M protein of SARS-CoV was also reported to ably interact with TBK1/IKK*ϵ* and TRAF3, which blocked the formation of a functional TRAF3.TANK.TBK1/IKK*ϵ* complex and lead to the failure of IRF3 phosphorylation (39).

**Figure 7:**
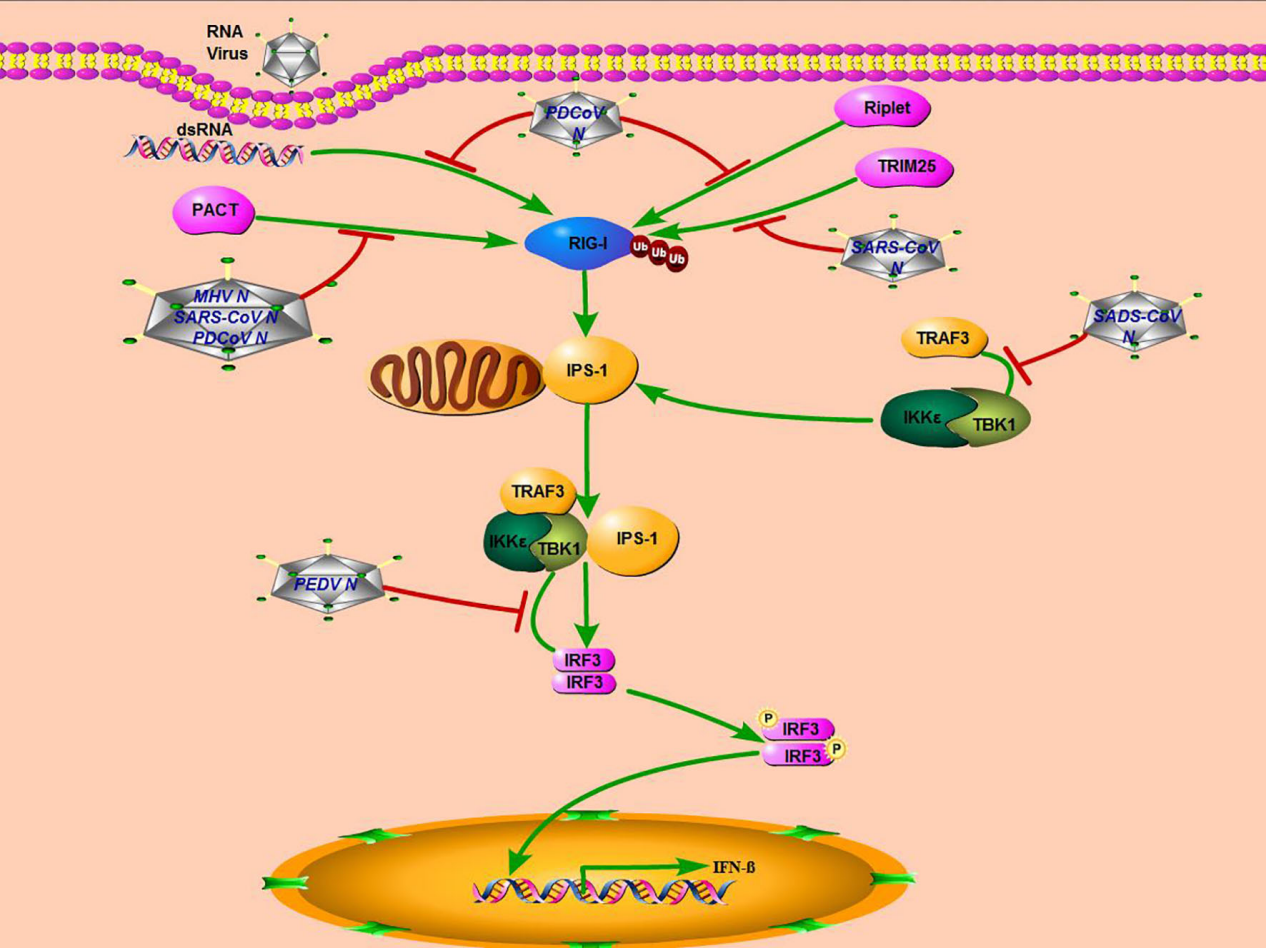
Mechanisms of coronavirus N protein inhibiting IFN-*β* production. SARS-CoV, MHV, and PDCoV inhibit interferon production by targeting molecules upstream of the RLR signaling pathway (RIG-I), while PEDV and SADS-CoV, which belong to the genus Alphacoronavirus, inhibit interferon production by targeting molecules downstream of the RLRs signaling pathway (TBK1).

The N protein is important for coronavirus replication and proliferation by influencing host cellular responses, especially the antiviral immune response (40–42). The molecule mechanisms adopted by the N protein of some coronaviruses, including MERS-CoV, SARS-CoV, PEDV, PDCoV, MHV, and SADS-CoV in this study, were summarized in **Figure 7**. Although SADS-CoV and PEDV belong to the same genus of *Alphacoronavirus* and N proteins encoded by them, both can interact with TBK1, but their mechanisms of inhibiting IFN-*β* production are different. PEDV N protein targeted the downstream of the IPS-1–TBK1–IRF3 signal axis and disrupted the interaction between IRF3 and TBK1 to antagonize IFN-*β* production (18). Previous research reported that the N proteins encoded by two beta coronavirus MHV and SARS-CoV, and one delta coronavirus PDCoV all block the interaction between PKR activating protein (PACT) and RIG-I to depress RIG-I binding with dsRNA (20, 36).

It has also been reported that the SARS-CoV N protein antagonizes IFN-*β* production by inhibiting TRIM25-mediated RIG-I ubiquitination (43). And PDCoV N protein could also directly target RIG-I and suppress its early activation by interfering with dsRNA and Riplet-mediated K63-linked polyubiquitination (19). It seems that there are two primary ways for the coronavirus N protein to inhibit IFN-*β* production. One is that non-alphacoronavirus N protein could affect the RLRs’ signaling pathway upstream, blocking RIG-I recognition or activation. The other is that the N protein of alphacoronavirus, including SADS-CoV and PEDV, inhibits IFN-*β* expression by targeting TBK1, a downstream molecule of the RLRs’ signaling pathway. More studies are needed to fully elucidate molecular mechanisms employed by the coronavirus N protein to antagonize the host innate immune response.

We also found that SADS-CoV N protein interacted not only with TBK1 but also with IKK*ϵ*. TBK1 and IKK*ϵ* are two key kinases with similar structure and function. The difference between them is that TBK1 is widely expressed in various types of cells, while IKK-*ϵ* is chiefly expressed in lymphoid cells and inducible in other cell types (44, 45). Furthermore, TBK1 is mainly involved in the phosphorylation of IRF3 and IRF7 and the subsequent antiviral reactions, whereas IKK-*ϵ* is assisted, which suggests that TBK1 plays a more important role than IKK-*ϵ* (44, 46, 47). Congruent with these findings, our luciferase reporter gene assay revealed that the activity of IFN-*β* promoter induced by TBK1 was more significant than that induced by IKK-*ϵ*, as well as that the inhibitory effect of SADS-CoV N protein on the activation of IFN-*β* promoter induced by TBK1 was more obvious, indicating that the interaction between SADS-CoV N protein and TBK1 acted a vital part in the virus escaping from innate immunity. However, attention on the interaction between SADS-CoV N protein and IKK-*ϵ* is still needed, because N protein’s simultaneous effect on TBK1 and IKK-*ϵ* may be more beneficial for SADS-CoV to infect different cells.

In summary, this study investigated the role of SADS-CoV N protein in the host innate immunity. And the results showed that SADS-CoV N protein obstructed interactions between TRAF3 and TBK1 to make TBK1 activation fail, which in turn led to reduced IFN-*β* production. Our results have enriched mechanisms utilized by the coronavirus N protein. Future research of functions for other SADS-CoV proteins is warranted to understand better the evasion strategies through which SADS-CoV antagonizes the antiviral immune response.

## Data Availability Statement

The original contributions presented in the study are included in the article/supplementary material. Further inquiries can be directed to the corresponding authors.

## Author Contributions

JM conceived and designed the study and critically revised the manuscript. ZZ and YS wrote the manuscript. ZZ performed the experiments and conducted data analysis. JX, XT, LZ, QL, and TL helped in experimental implementation. All authors contributed to the article and approved the submitted version.

## Conflict of Interest

The authors declare that the research was conducted in the absence of any commercial or financial relationships that could be construed as a potential conflict of interest.
